# Brazilian Amazon Red Propolis: Leishmanicidal Activity and Chemical Composition of a New Variety of Red Propolis

**DOI:** 10.3390/metabo13091027

**Published:** 2023-09-21

**Authors:** Richard Pereira Dutra, Marcos Marinho de Sousa, Maria Simone Pereira Maciel Mignoni, Karla Gabriela Mota de Oliveira, Euzineti Borges Pereira, Aline Santana Figueredo, Arthur André Castro da Costa, Tatielle Gomes Dias, Cleydlenne Costa Vasconcelos, Lucilene Amorim Silva, Aramys Silva Reis, Alberto Jorge Oliveira Lopes

**Affiliations:** 1 Laboratory of Natural Products Chemistry, Federal University of Maranhão, Imperatriz 65915-240, Brazil; 2 Program in Health and Technology, Federal University of Maranhão, Imperatriz 65915-240, Brazil; 3 Laboratory of Pathology and Immunoparasitology, Federal University of Maranhão, São Luís 65080-805, Brazil; 4 Biological and Health Sciences Center, Federal University of Maranhão, Av. dos Portugueses 1966, São Luís 65085–580, Brazil; 5 Chemistry Postgraduate Program, Federal Institute of Science Education and Technology of Maranhão, São Luís 65030-005, Brazil

**Keywords:** red propolis, isoflavonoids, leishmanicidal activity, natural products, new drug agents, medicinal chemistry

## Abstract

Leishmaniasis is caused by protozoans of the genus *Leishmania*, and its treatment is highly toxic, leading to treatment discontinuation and the emergence of resistant strains. In this study, we assessed the leishmanicidal activity and chemical composition of red propolis collected from the Amazon-dominated region of northern Tocantins State, Brazil. The MTT assay was employed to determine the samples’ activity against *Leishmania amazonensis* promastigotes and their cytotoxicity against RAW macrophages. Spectrophotometric assays were utilised to measure the concentrations of total phenolics and flavonoids, while high-performance liquid chromatography coupled to a mass spectrometer (LC-MS/MS) was used to determine the chemical composition. An in silico study was conducted to evaluate which compounds from Brazilian Amazon red propolis may correlate with this biological activity. Brazilian Amazon red propolis exhibited a high concentration of phenolic compounds and an inhibitory activity against *L. amazonensis*, with an IC_50_ ranging from 23.37 to 36.10 µg/mL. Moreover, fractionation of the propolis yielded a fraction with enhanced bioactivity (16.11 µg/mL). Interestingly, neither the propolis nor its most active fraction showed cytotoxicity towards macrophages at concentrations up to 200 µg/mL. The red colour and the presence of isoflavonoid components (isoflavones, isoflavans, and pterocarpans) confirm that the substance is Brazilian red propolis. However, the absence of polyprenylated benzophenones suggests that this is a new variety of Brazilian red propolis. The in silico study performed with two of the main leishmanicidal drug targets using all compounds identified in Amazon red propolis reported that liquiritigenin was the compound that exhibited the best electronic interaction parameters, which was confirmed in an assay with promastigotes using a standard. The findings indicate that Amazon red propolis possesses leishmanicidal activity, low toxicity, and significant biotechnological potential.

## 1. Introduction

Propolis is a natural product produced by bees of the genus *Apis*, formed by combining resinous material collected from buds, flower buds, and plant exudates with enzymatic salivary secretions and beeswax [1]. In Brazil, propolis produced by *Apis mellifera* L. is classified based on its physicochemical properties, chemical composition, and botanical origin. The thirteenth type, known as red propolis, is derived from the resin of *Dalbergia ecastaphyllum* (L.) Taub. (Fabaceae) found in mangrove areas and *Symphonia globulifera* L.f. (Clusiaceae) found in the Atlantic Forest, in biomes along the north-eastern coast of Brazil [2].

Red propolis from north-eastern Brazil exhibits various biological activities, such as antibacterial [3,4], antifungal [4], antioxidant [4,5], antitumor [4], anti-inflammatory, immunomodulatory [6], cytotoxic [5], anticancer [7], and antiprotozoal [8,9]. These activities are attributed to its chemical composition, which includes isoflavonoids, chalcones, tannins, catechins, flavones, biflavonoids, flavonols, pentacyclic triterpenoids, xanthones, and polyprenylated benzophenones [2,10]. However, it is essential to note that the chemical composition and biological activities of red propolis can vary depending on its geographical origin [1]. Interestingly, while red propolis from the north-eastern coast of Brazil has been extensively studied, to date, there are no reports of the production of this propolis in other regions, including the Amazon region.

The Amazon biome, spanning multiple states in northern Brazil, including Tocantins, is known for its incredible biodiversity [11]. Its flora is so rich and diverse that it is reflected in the unique chemical composition of its propolis. Ishida et al. [12] discovered a new type of propolis in the Brazilian Amazon region containing prenylated benzophenones, which have shown strong antibacterial properties. Additionally, propolis from *A. mellifera* produced in another region of the Amazonian biome was found to contain 87 compounds, mainly comprising monoterpenes, sesquiterpenoids, and phenylpropanoids [13]. This chemical diversity of Amazonian propolis is of particular interest in the development of new drugs for neglected diseases such as leishmaniasis.

Leishmaniasis is a group of parasitic diseases caused by protozoa of the genus *Leishmania* and transmitted to humans by sandflies of the genus *Phlebotomus* and *Lutzomyia.* This disease affects millions of people worldwide and can manifest in various forms [14,15]. Unfortunately, there is currently no effective vaccine, and the available drugs have limited efficacy and several side effects [15]. Consequently, there is an urgent need to develop new drugs for the treatment of leishmaniasis, and natural products have shown great promise in this regard, especially those produced by bees. In a recent study, we reported the leishmanicidal activity of geopropolis extract produced by *Melipona fasciculata* Smith 1858 (Apidae, Meliponini), highlighting the potential of bee products in identifying candidate molecules for treating leishmaniasis [16].

In this study, we evaluated the leishmanicidal activity and chemical composition of a new variety of Brazilian red propolis collected in the Amazon biome in northern Tocantins State, Brazil. The unique chemical composition of this new propolis variety offers a new pathway for the development of more effective treatments for leishmaniasis. Our findings not only demonstrate the leishmanicidal activity of this new propolis variety but also highlight its potential as a source of novel bioactive compounds for the treatment of leishmaniasis.

## 2. Materials and Methods

### 2.1. Collecting Propolis and Obtaining Extracts

**Figure 1:**
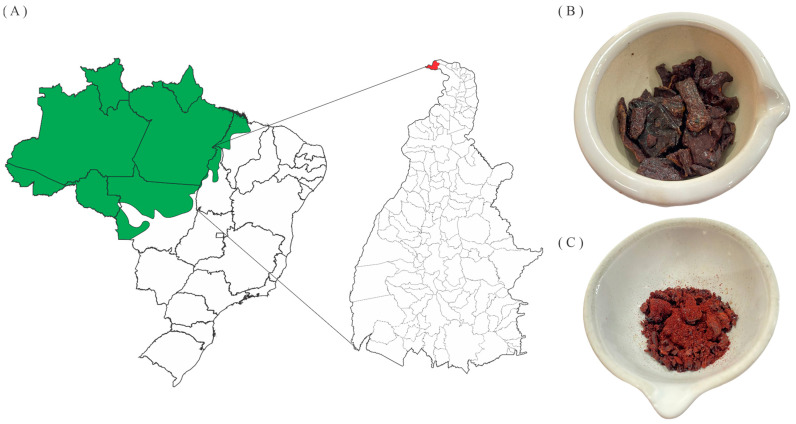
(**A**) Location of the Amazon Biome (in green), including the State of Tocantins and the city of Esperantina (in red); (**B**) raw red propolis; (**C**) dry hydroethanolic extract of red propolis from the Brazilian Amazon.

Four samples of red propolis were collected between 2017 and 2019 in the Amazon region of the municipality of Esperantina, Tocantins State, Brazil (5°23′12.3″ S, 48°33′20.6″ W) (Figure 1A). The samples (Figure 1B) were macerated in 70% ethanol for 48 h, in a proportion of 1:5 mass-to-volume (*m*/*v*) ratio of propolis and solvent, followed by vacuum filtration and concentration in a rotary evaporator under a vacuum (Quimis, model Q34432) at 45 °C to obtain the red propolis hydroethanolic extracts (EHPV 1–4) (Figure 1C). EHPV-1 (3 g) was fractionated by a liquid–liquid partition with organic solvents of progressively increasing polarity: hexane (FrHX), chloroform (FrCL), and ethyl acetate (FrEA). The extraction yield was expressed as a proportion of the mass ratio of propolis to dry extract [16]. The yields of Brazilian Amazon red propolis hydroethanolic extracts (EHPV 1–4) ranged from 19.56 to 64.40% by mass, while the fractions obtained from the extract (EHPV-1) showed a yield of 76.67% (2.30 g) for the chloroform fraction (FrCL), followed by the ethyl acetate fraction (FrEA) with 11.60% (348 mg) and the n-hexane fraction (FrHX) with 9.97% (299 mg).

### 2.2. Determining the Total Concentrations of Phenolics and Flavonoids

Extracts and fractions were dissolved in methanol at a concentration of 2.0 mg/mL. The total phenolic concentration was determined through the reaction of 0.1 mL of the samples with 1.0 mL of 20% sodium carbonate (Isofar, Rio de Janeiro, RJ, Brazil), followed by 0.1 mL of Folin-Ciocalteau reagent (Sigma-Aldrich, São Paulo, SP, Brazil), resulting in a final volume of 4.0 mL in distilled water. After homogenization, the samples were incubated for 2 h in the absence of light. Absorbance readings were taken at 760 nm, and the results were expressed in milligrams equivalent to gallic acid per gram of the sample (mg GAE/g), using a gallic acid calibration curve (Sigma-Aldrich, São Paulo, SP, Brazil) in the range of 0.0025–0.04 mg/mL (r^2^ = 0.998). The concentrations of total flavonoid were determined by reacting 0.5 mL of the samples with 0.5 mL of a 5% methanolic solution of aluminum chloride hexahydrate (Isofar, Rio de Janeiro, RJ, Brazil), resulting in a final volume of 4.0 mL in methanol. Subsequently, the samples were homogenised, and after 30 min of the reaction, under light protection, absorbances were obtained at 425 nm. The results were expressed as milligrams equivalent to quercetin per gram of the sample (mg QE/g), based on a quercetin calibration curve (Sigma-Aldrich, São Paulo, SP, Brazil) with concentrations ranging from 0.001 mg/mL to 0.02 mg/mL (r^2^ = 0.999). For both analyses, absorbances were measured in triplicate using a UV-Vis spectrophotometer (Gehaka, UV-340G, São Paulo, SP, Brazil) [17,18].

### 2.3. Antileishmanial Activity

The leishmanicidal activity against axenic cultures of *Leishmania* (*Leishmania*) *amazonensis* promastigotes (MHOM/BR/1987/BA-125) grown in Schneider’s medium was evaluated (Sigma-Aldrich, São Paulo, Brazil). Propolis extracts and fractions (200–12.5 µg/mL) or compounds—biochanin A, liquiritigenin, isoliquiritigenin, calycosin, and formononetin—(15.60, 7.81, and 3.90 µg/mL) were solubilised in Schneider’s medium in 96-well plates containing a final volume of 100 µL and 5 × 10^6^ parasites per well. Amphotericin B was used as a positive control. Negative controls included culture medium, 1% ethanol, or 1% dimethyl sulfoxide (DMSO) in culture medium [19]. After 24 h of incubation, 10 µL of a 5 mg/mL solution of MTT (3-(4,5-dimethylthiazol-2-yl)-2,5-diphenyltetrazolium bromide) was added to determine promastigote viability. Following an overnight reaction, 100 µL of 10% SDS 0.01N HCl was added to dissolve the formazan crystals. Absorbances were measured at 550 nm using a microplate reader (Biotek ELx800, Winooski, VT, USA).

### 2.4. Cytotoxicity Assay

Cytotoxicity was evaluated against the murine nontumor cell line RAW-264.7 (murine macrophage-ATCC: TIB-71). Initially, to promote cell spreading and adhesion, RAW 264.7 cells (5 × 10^4^ cells/well) were cultured in 96-well plates and incubated with 100 μL of RPMI 1640 medium supplemented with foetal bovine serum (10%), penicillin (100 U/mL), streptomycin (100 μg/mL), amphotericin B (2.5 μg/mL), L-glutamine (2 mmol/L), and sodium pyruvate (1 mmol/L) in a 37 °C atmosphere containing 5% CO_2_ and 95% humidity for 12 h. Afterward, 100 µL of serial dilutions of propolis extracts and fractions (200 to 6.75 μg/mL) were added to the supplemented RPMI medium for 24 h in a 37 °C atmosphere containing 5% CO_2_ and 95% humidity. Following this period, 10 µL of MTT solution at a concentration of 5 mg/mL was added and allowed to react for 3 h in the absence of light; then, formazan crystals were solubilised using DMSO. Absorbance readings were taken at 550 nm in a microplate reader (Biotek Elx800, Winooski, VT, USA) [20].

### 2.5. Liquid Chromatography-Tandem Mass Spectrometry (LC-MS/MS)

EHPV-1 and FrCL were analysed using a Shimadzu HPLC-SCL-10A (Kyoto, Japan) equipped with two pumps (LC-10AD) and an automatic injector, and coupled to a mass spectrometer (Amazon Speed ETD, Bruker, Billerica, MA, USA) equipped with electrospray ionization (ESI) and an ion-trap-type analyser. The samples were dissolved in HPLC-grade methanol (5 mg/mL), filtered through a 0.22 µm Nylon syringe filter (Allcrom, São Paulo, SP, Brazil), and injected into a C-18 reverse-phase analytical column (250 × 4.6 mm, 5 µm, Phenomenex^®^ Luna, Torrance, CA, USA) at a flow rate of 1.0 mL/min and detection at 280 nm at room temperature (25 °C). The mobile phases were ultrapure water containing 0.1% formic acid (A) and methanol (B) with the following linear gradient: 1 min, 5% B; 5 min, 30% B; 15 min, 40% B; 55 min, 50% B; 80 min, 60% B; 90 min, 100% B. The mass spectra were obtained in the positive mode, with a capillary voltage of 4.5 kV and temperature of 300 °C, using nitrogen as the nebulization gas at low resolution, and in the scan mode with an *m/z* range from 100 to 1500. The compounds were identified by comparing their molecular ion mass, fragmentation patterns, and mass spectra from the literature, as well as through co-elution with analytical standards (HPLC standards, ≥95%) purchased from Sigma-Aldrich (São Paulo, SP, Brazil) for biochanin A, formononetin, isoliquiritigenin, and liquiritigenin. Furthermore, calycosin was obtained through chromatographic isolation and identified via nuclear magnetic resonance (NMR) spectroscopic analysis by our research group [17]. The relative percentage of each identified compound was calculated using DataAnalysis 4.3 software (Compass DataAnalysis 4.3 Bruker, UK). 

### 2.6. In Silico Analysis

The compounds identified in EHPV-1 were structurally represented in 3D using the GaussView^®^ 5.0.8 program [21]. Geometric and vibrational properties were calculated and optimised in a vacuum at the Density Functional Theory (DFT) level, employing the hybrid functional B3LYP combined with the 6–31 ++ G (d, p) basis set in Gaussian^®^ 16 [22].

The sterol 14-alpha-demethylase (CYP51) and trypanothione reductase (TR) of *L. infantum* (99.3% and 95.3% similarity with *L. amazonensis*, respectively) were obtained from the Protein Data Bank (PDB) (#3L4D; #2W0H). Fluconazole, antimony, and other molecules in the crystal structures were removed, retaining only one of the two homologous chains and the HEME group (only in CYP51).

Molecular docking (MD) procedures were performed using Autodock Vina^®^ [23]. The structures and ligands were prepared for MD calculations employing AutoDock^®^ Tools 1.5.7 [24]. The MD methodology described in the literature [25] was adopted, with modifications [18]. Gasteiger partial charges were calculated after adding all hydrogens for both ligands and target structures. The cubic box dimensions on the X, Y, and Z axes were 30 × 30 × 30. The grid box was centred on the iron atom of the CYP51 HEME group and the sulphur atom of the Cys52 amino acid residue in TR. The number of modes was set to 50, and the exhaustiveness was set to 24. The conformations with the best interaction energy for the ‘ligand + receptor’ complexes identified in molecular docking were selected based on the free energy of binding, visual inspection, and analysis of residues that interacted optimally with the ligand. Molecular analyses and complex representations were obtained using the UCSF Chimera package [26] and PoseView [27]. Likewise, the PDB structures were redocked with the original ligand using the same conditions and protocols as those used with the identified compounds, and the root mean square deviation (RMSD) of the ligand position predicted by docking with the original position of the ligand in the crystal was evaluated using the Swiss PDB-Viewer.

### 2.7. Statistical Analysis

The results for total phenolic and flavonoid concentrations were expressed as mean ± standard deviation, based on triplicate analyses. For the determination of the inhibitory concentration (IC_50_), data from dose-response experiments were fitted to a non-linear regression model using the sigmoidal dose-response (variable slope) equation. This method provides an accurate estimation of the IC_50_ value, which represents the concentration of the compound required to achieve a 50% inhibition. Analysis of variance (ANOVA) with Tukey’s multiple comparison test was applied to assess significant differences between means, with a reliability level of 5% (*p* < 0.05). All analyses were conducted using GraphPad Prism software (Version 8.0 for Windows, San Diego, CA, USA).

## 3. Results

### 3.1. Total Phenolic Content and Total Flavonoids Content

The propolis samples exhibited an intense red colour, with total phenolic concentrations in the extracts ranging from 192.03 ± 7.06 to 336.91 ± 8.77 mg GAE/g, while the flavonoid concentrations ranged from 16.70 ± 0.88 to 34.38 ± 0.44 mg QE/g. These results indicate that propolis collected from the same location may contain varying amounts of its constituents.

Statistical analysis revealed significant variation in the concentration of total phenolics among all extracts (EHPV 1–4), whereas no significant difference was observed between EHPV-2 and EHPV-4 for flavonoids among EHPV-1, EHPV-2, and EHPV-3, indicating greater variation in total phenolic concentration. The EHPV-1 extract was fractionated with organic solvents to obtain fractions with different total phenolic concentrations, with the highest concentration for the chloroform fraction (FrCL) at 328.44 ± 7.51 mg GAE/g, a concentration 44% higher than that obtained in the ethyl acetate fraction (FrEA) and 76% higher than that obtained in the n-hexane fraction (FrHX). However, there was no significant difference in flavonoid concentration for the fractions FrCL and FrEA, and flavonoids were not detected in FrHX. The fractionation facilitated separation based on the solvent type, with compounds exhibiting higher affinity for medium-polarity solvents (chloroform) (Table 1).

### 3.2. Leishmanicidal Activity

Extracts and fractions of Brazilian Amazon red propolis were classified as highly active (IC_50_ < 10 µg/mL), active (IC_50_ > 10 < 50 µg/mL), moderately active (IC_50_ > 50 < 100 µg/mL), and non-active (IC_50_ > 100 µg/mL) [28]. All extracts demonstrated activity against *L. amazonensis* promastigotes, with IC_50_ values ranging from 23.37 to 36.10 µg/mL. The most active extract was EHPV-1, which exhibited a significant difference from EHPV-3. The FrCL fraction showed the highest promastigote inhibition; however, there was no significant difference compared to EHPV-1. The concentrations at which FrHX and FrEA exhibited inhibition indicate that the fractionation was selective in obtaining active fractions, with the most polar fraction (FrEA) being inactive (Table 2).

**Figure 2:**
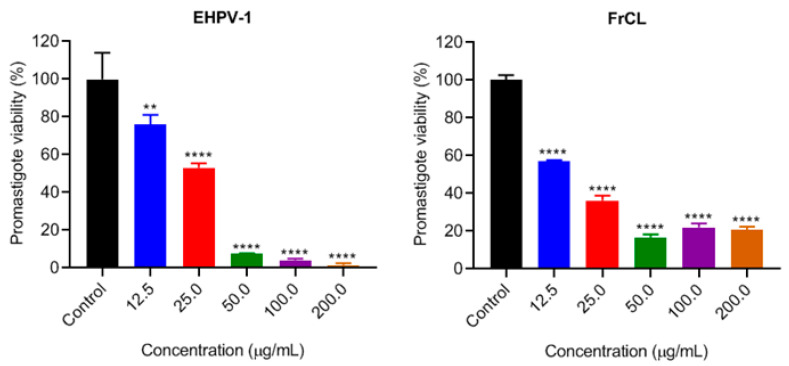
Inhibition of *L. amazonensis* promastigotes proliferation by the hydroalcoholic extract (EHPV-1) and chloroform fraction (FrCL) of Brazilian Amazon red propolis in vitro. Data represent means ± SD of triplicate cultures. ** *p* < 0.01 or **** *p* < 0.0001 compared to the control group.

EHPV-1 and FrCL demonstrated a dose-dependent inhibition of *L. amazonensis* promastigotes, with an inhibition from approximately 92 to 98% of promastigotes at the three highest concentrations tested. In comparison, FrCL inhibited 80% of promastigotes at the same concentrations, indicating a synergistic effect of the compounds in the extract (Figure 2).

### 3.3. Cytotoxicity in Raw 264.7 Cells and Selective Index

**Figure 3:**
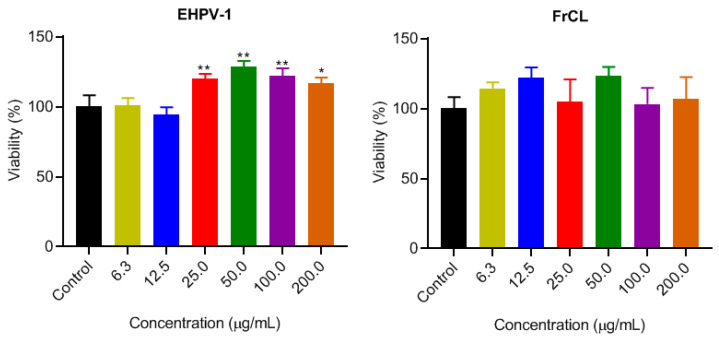
Cytotoxicity against RAW 264.7 macrophages after treatment with hydroalcoholic extract (EHPV-1) and chloroform fraction (FrCL) of Brazilian Amazon red propolis in vitro. Data represent means ± SD of triplicate cultures. * *p* < 0.05 or ** *p* < 0.01 compared to the control group.

The MTT assay was conducted to assess the cytotoxicity of the EHPV-1 extract and FrCL fraction on normal RAW 264.7 cells. These cells play a pivotal role in controlling infection within the immune system. The results revealed that both EHPV-1 and FrCL were non-cytotoxic at the tested concentrations. Furthermore, higher concentrations of EHPV-1 promoted cell proliferation (Figure 3). Consequently, it was not possible to calculate the IC_50_. To assess the potential for leishmaniasis treatment, we calculated the selectivity index (SI) by determining the ratio between the cellular cytotoxicity (CC_50_) of macrophages and the IC_50_ of leishmaniasis. An SI greater than 1 suggests the potential for treating leishmaniasis [29]. Interestingly, both the extract and the fraction had a selectivity index greater than 8.6 and 12.4, respectively.

### 3.4. The Chemical Composition of Brazilian Amazon Red Propolis

**Figure 4:**
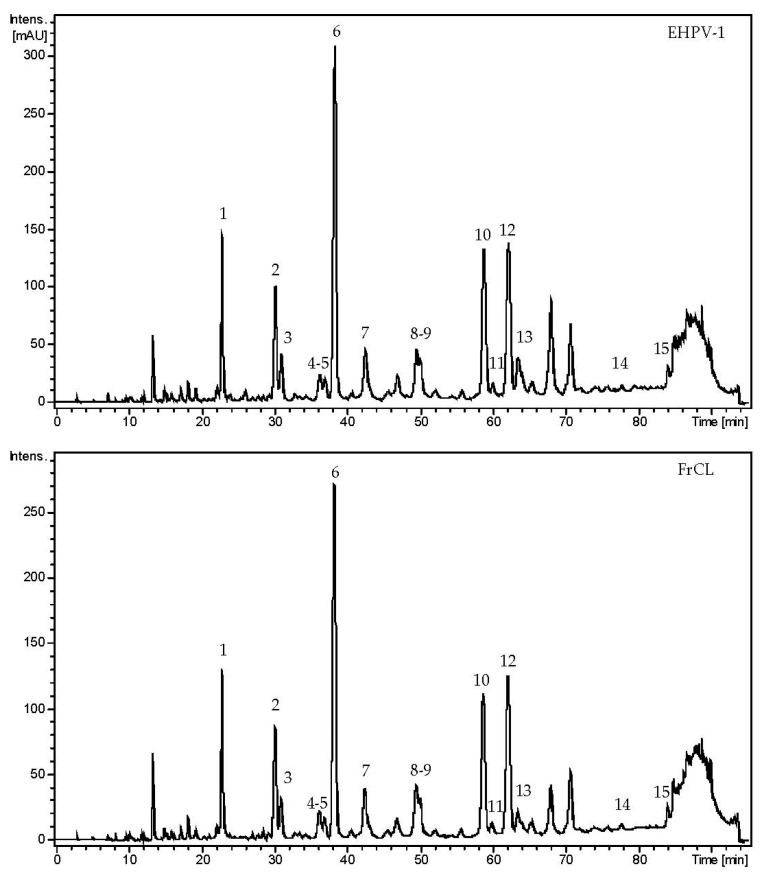
Chromatographic profiles obtained at 280 nm by LC-MS/MS of the hydroalcoholic extract (EHPV-1) and chloroform fraction (FrCL) of the Brazilian Amazon red propolis.

**Figure 5:**
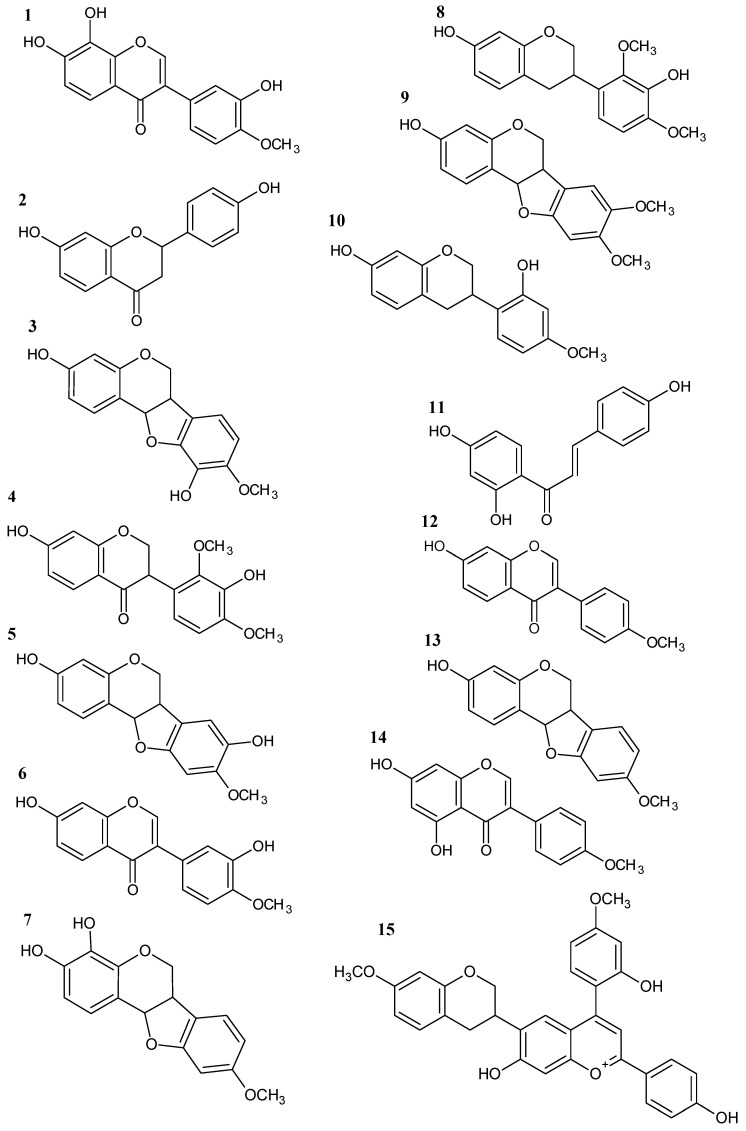
Structures of phenolic compounds identified in Brazilian Amazon red propolis by LC-ESI-MS/MS: 7,8,3′-trihydroxy-4′-methoxyisoflavone (**1**); liquiritigenin (**2**); vesticarpan (**3**); violanone (**4**); 3,8-dihydroxy-9-methoxy-pterocarpan (**5**); calycosin (**6**); 3,4-dihydroxy-9-methoxy-pterocarpan (**7**); mucronulatol (**8**); 3-hydroxy-8,9-dimethoxy-pterocarpan (**9**); vestitol (**10**); isoliquiritigenin (**11**); formononetin (**12**); medicarpin (**13**); biochanin A (**14**); retusapurpurin B (**15**).

A total of 15 flavonoids were tentatively identified in EHPV-1 and FrCL by LC-MS/MS. The main chemical class was isoflavonoids, with four isoflavones (7,8,3′-trihydroxy-4′-methoxyisoflavone, calycosin, formononetin, and biochanin A), two isoflavans (vestitol and mucronulatol), five pterocarpans (vesticarpan, 3,8-dihydroxy-9-methoxy-pterocarpan, 3,4-dihydroxy-9-methoxy-pterocarpan, 3-hydroxy-8,9-dimethoxy-pterocarpan, and medicarpin), as well as a flavanone (liquiritigenin), a isoflavanone (violanone), a chalcone (isoliquiritigenin), and a chalcone-condensed isoflavone (retusapurpurin B). The chromatographic profiles indicate that the two main compounds were calycosin, accounting for 21.87% in EHPV-1 and 23.78% in FrCL, and formononetin, with a proportion of 12.72% in EHPV-1 and 14.42% in FrCL, as demonstrated in Figure 4. Table 3 summarises the precursor ions along with their corresponding fragmentations. Figure 5 shows the proposed structures for the chemical compounds of Brazilian Amazon red propolis.

### 3.5. In Silico Study

For our molecular docking analysis, we used all compounds identified by LC-MS/MS in the hydroethanolic extract (EHPV-1). In general, all compounds identified in the Brazilian red propolis extract showed the highest affinity parameters for the selected *Leishmania* targets. Liquiritigenin was the compound that exhibited the best electronic affinity parameters, with a free energy binding value of −9.3 kcal/mol for sterol 14-alpha-demethylase (CYP51) and −8.9 kcal/mol for trypanothione reductase (TR). Similarly, we highlight the values obtained by calycosin (also −9.3 kcal/mol) for CYP51 and 7,8,3′-trihydroxy-4′-methoxyisoflavone (−8.2 kcal/mol) for TR (Table 4).

**Figure 6:**
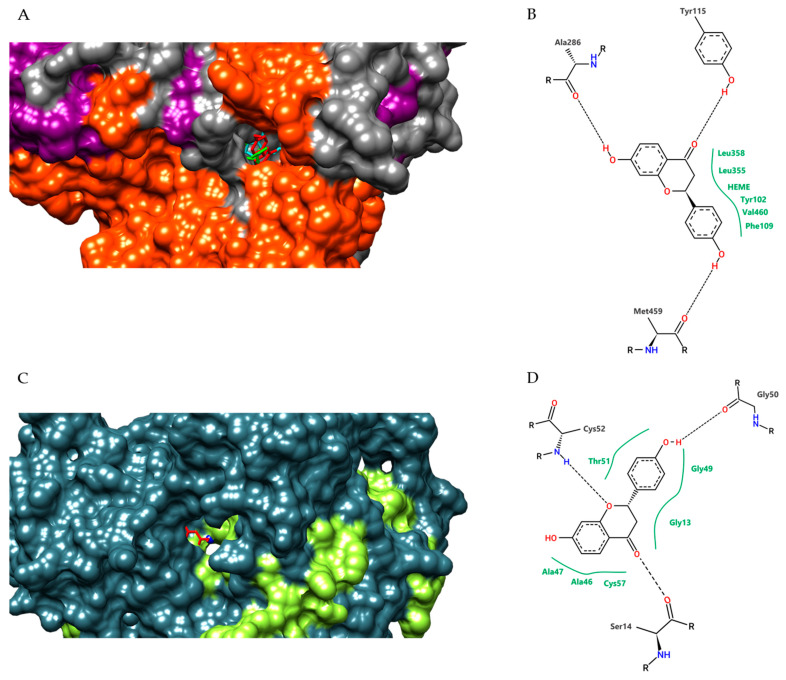
Detail representation in the surface format of conformations obtained by molecular docking of liquiritigenin (red), and fluconazole (green); HEME group (cyan) at the LiCYP51 active site (**A**); and liquiritigenin (red), and antimony (blue) at TR active site (**C**); two-dimensional diagram of the interactions performed by liquiritigenin with the amino acid residues and HEME group of the LiCYP51 active site (**B**) and with the amino acid residues of TR (**D**). Dashed black lines represent hydrogen bonds, while full green lines represent van der Waals interactions.

Evaluating the interaction of the conformation with the lowest binding free energy of the complexes formed by liquiritigenin with LiCYP51 and TR, we observed that this flavonoid formed stable interactions with amino acid residues of the active site of both enzymes, involving hydrogen bonding with residues Tyr115, Ala286, and Met459, and van der Waals contacts with Tyr102, Phe109, Leu355, Leu358, Val460, and the HEME group for LiCYP51. For TR, hydrogen bonds were formed with residues Ser14, Gly50, and Cys52, and van der Waals contacts with Gly13, Ala46, Ala47, Gly49, Thr51, and Cys57 (Figure 6).

### 3.6. Antipromastigote Activity of Flavonoids from Amazon Red Propolis

**Figure 7:**
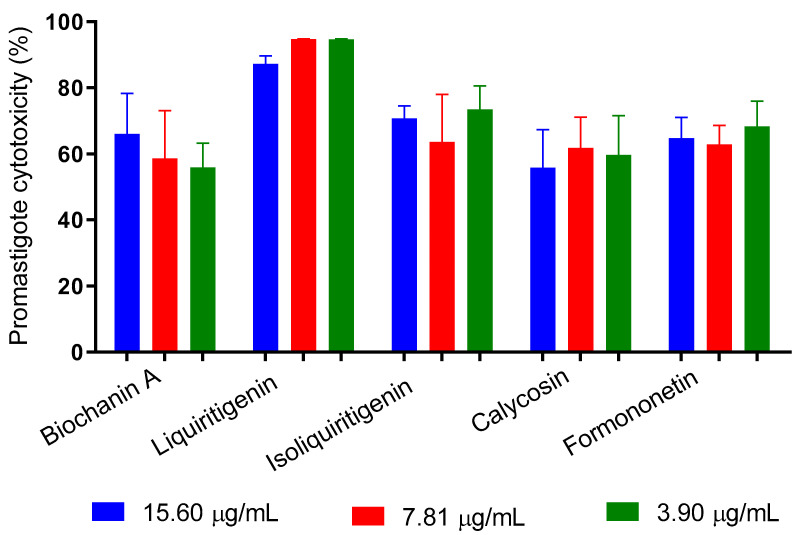
In vitro inhibition of the proliferation of promastigotes of *L. amazonensis* by flavonoids identified in Brazilian Amazon red propolis.

We tested the antipromastigote effect of five flavonoids identified in Brazilian Amazon red propolis. The results indicate that at all tested concentrations (15.60 to 3.90 μg/mL), the substances promoted cytotoxicity greater than 50%, with no dose-dependent effect. Liquiritigenin had the best activity and induced death in 87.2% to 94.7% of the promastigotes (Figure 7).

## 4. Discussion

It is well-established that the chemical composition and biological activities of propolis are related to the botanical source utilised by bees and the geographical location [4]. Here, for the first time, we performed the chemical and biological characterization of a new red propolis variety, which we call Brazilian Amazon red propolis. We identified this red propolis in the Amazon region of the State of Tocantins, Brazil. Interestingly, it has a different chemical profile from the red propolis from north-eastern Brazil and potent antileishmanial activity.

Initially, we determined the concentration of total phenolic compounds and flavonoids. The determination of total phenolic concentration by Folin-Ciocalteau quantifies phenolic acids, flavonoids, and tannins, and the flavonoid assay (AlCl_3_) is selective for flavonoids, thereby reducing the interference of phenolic acids typically present in propolis. According to the Brazilian Ministry of Agriculture, these tests should be utilised to determine the quality of propolis [30]. Notably, Amazon red propolis has a higher concentration of compounds than red propolis from the Northeast. Using Folin-Ciocalteau, Brazilian red propolis from Alagoas contained 7.33 mg GAE/g of total phenolics, while propolis from Sergipe contained 151.55 mg GAE/g. These phenolic compound concentrations have been linked to antioxidant and antibacterial activities [3,31]. Here, Amazon red propolis has between 192.03 and 336.91 mg GAE/g of total phenolics.

Additionally, we showed higher concentrations of total phenolics in the chloroform fractions, followed by the ethyl acetate and hexane fractions. A similar profile has been shown for red propolis from Alagoas State, Brazil, which also exhibits higher concentrations of total phenolics in the chloroform fractions. Red propolis produced in Northeastern Brazil has been demonstrated in studies to exhibit leishmanicidal activity against various species of the genus *Leishmania*. The results of the current study were corroborated by Araújo et al. [32], who used hydroethanolic extracts of red propolis made in Sergipe State, Brazil from the botanical source *D. ecastaphyllum*, which had an IC_50_ of 9.73 μg/mL for promastigotes of *L. amazonensis* and 21.54 μg/mL for *L. chagasi*.

In comparison to brown and green propolis, Brazilian red propolis from Bahia State demonstrated greater leishmanicidal activity in a macrophage (BALB/c) infection test with *L.* (*V*) *braziliensis* [8]. The authors attributed this activity to the presence of phenolic compounds and antioxidant activity.

Leishmanicidal assays with red propolis from Pernambuco and Alagoas, Brazil inhibited promastigotes of *L. braziliensis* and *L. infantum*, with greater susceptibility to *L. braziliensis*. The propolis from Pernambuco collected in the rainy season was more active, with an IC_50_ of 35.22 and 37.45 μg/mL for *L. braziliensis* and *L. infantum*, respectively. The study demonstrated the action of *D. ecastaphyllum* resins, with an IC_50_ of 90.46 μg/mL (*L. braziliensis*) and 134.56 μg/mL (*L. infantum*), indicating chemical alteration during the production of propolis by bees. Moreover, the level of inhibition was different depending on the type of *Leishmania*, the time of year, and the place where the propolis was collected [9].

The production of polymeric nanoparticles with red propolis from Alagoas, Brazil was efficient in encapsulating the flavonoids, producing a material with controlled release against *L. braziliensis*; with antioxidant activity, it is more active than the extract [33,34]. These studies indicate the potential of Brazilian red propolis for the treatment of leishmaniasis.

Assessing the cytotoxic potential of drug candidates in vitro is a critical step in the development of safe therapeutics. If extracts or substances reduce viability to less than 70% of the control, they exhibit cytotoxic potential (ISO 10993-5:2009). In this study, we evaluated the toxicity of EHPV-1 and FrCL at concentrations up to 200 μg/mL and found that neither extract was toxic. In fact, higher concentrations of these extracts even induced cell proliferation. In vitro cell viability assays in murine macrophages demonstrated that concentrations from 160 µg/mL of ethanolic extracts of red and green propolis affected cell viability [8].

Red propolis extracts evaluated against mouse fibroblast cell lines (NCTC929) demonstrated that concentrations lower than 31.25 μg/mL do not possess toxicity [9]. A viability study with non-tumour VERO (monkey kidney epithelial) cells, using the MTT assay, demonstrated that extracts and subfractions of red propolis from Alagoas (Brazil) showed a cytotoxic effect. These authors suggest that to ensure safety in the use of red propolis, one should use concentrations lower than the minimum inhibitory concentration [3]. These data show that Amazon red propolis presents low cytotoxicity for macrophages, indicating its safe use in leishmanicidal assays.

The results obtained by LC-MS/MS demonstrate that Amazon red propolis, produced in the State of Tocantins, Brazil, has isoflavonoids as the main compounds. The chromatographic profiles of the extracts and fractions were obtained at 280 nm due to the intense absorption band of this compound class in the range of 245–290 nm (band II) with limited absorption above 300 nm (band I), resulting from the absence of conjugations between rings A and B. Electrospray ionization in a positive mode enabled the structural elucidation of compounds through retro-Diels–Alder (RDA) reactions. This can be observed in the product ions at *m/z* 137 and 123, indicating cleavage and the presence of a hydroxyl group in ring A. Similarly, ions at *m/z* 193, 181, 167, and 163 suggest fragments associated with ring B, which are characteristic of isoflavan and isoflavanone. Pterocarpans displayed ions with *m/z* 191, 161, and 153, which are also indicative of different RDA reactions. The ions at *m/z* 239, indicating the loss of the hydroxyl group attached to carbon 7 of ring A, followed by the ion at *m/z* 211, representing the loss of the carbonyl group (28 Da) from ring C, and finally, the ion at *m/z* 147, indicating the cleavage of ring B, allowed for the identification of flavonones and chalcones [31,35,36].

The isoflavones calycosin, formononetin, and biochanin A have been reported in Brazilian red propolis, produced near the mangroves of north-eastern Brazil, using the resins of *D. ecastaphyllum* [2,36]. Another identified isoflavone is hydroxy-calycosin (7,8,3′-trihydroxy-4′-methoxyisoflavone). This compound has been identified in the resin of *Amburana cearensis* (Allemão) A. C. Sm. (Fabaceae) [37] as well as in the propolis from Guinea-Bissau, where the authors suggest that the resin source is a species of the *Dalbergia* genus [36]. The isoflavans mucronulatol and vestitol have been reported in addition to Brazil, in red propolis from Cuba [35], Guinea-Bissau [38], and Nigeria [39]. The pterocarpans vesticarpan, 3,8-dihydroxy-9-methoxy-pterocarpan, 3,4-dihydroxy-9-methoxy-pterocarpan, 3-hydroxy-8,9-dimethoxy-pterocarpan, and medicarpin identified in this study, as well as the liquiritigenin, violanone, and isoliquiritigenin, are described for red propolis. Similarly, retusapurpurin B, a condensed isoflavone with a chalcone, is the substance reported in the literature as responsible for the red coloration of propolis and was identified in the resins of *Dalbergia* species [7,31,35].

The literature reports the compounds identified in this study in species of the Fabaceae family, tribe Dalbergieae, which suggests that the bees collected the resins from species of the genus *Dalbergia* [35,40]. A phytogeographic study was conducted near the area where the propolis used in this study was collected, and identified the presence of the plant species *Dalbergia monetaria* L.f. (Fabaceae) and *D. ecastaphyllum*, which corroborates our results [41].

These data corroborate the discovery of a new variety of Brazilian red propolis, considering that the presence of polyprenylated benzophenones characteristic of northeast propolis was not identified due to the use of *S. globulifera* resin [36]. The identification of isoflavonoids corroborates the leishmanicidal activity of red propolis in this study. The isoflavone calycosin (7,3′-dihydroxy-4′-methoxy isoflavone), the main constituent of the sample, has been reported to act against promastigote forms of *L. amazonensis* [42]. Calycosin showed selective toxicity to *Trypanosoma brucei* and *Leishmania donovani* amastigotes compared to Vero cells [43]. According to these authors, the chemical structure of isoflavones may be promising as antiprotozoal drugs.

Red propolis from Alagoas, Brazil presented the compounds liquiritigenin, isoliquiritigenin, formononetin, and biochanin A as chemical markers, with an IC_50_ of 38.3 μg/mL for promastigotes of *L. braziliensis* [33]. Inhibition of *L. donovani* promastigotes was observed for biochanin A with an IC_50_ of 2.5 μg/mL [44]. Among the possible mechanisms of the leishmanicidal action of flavonoids are the inhibition of arginase and the ability to chelate iron ions, reducing the availability of ribonucleotide reductase [45]. 

The presence of chalcones (isoliquiritigenin) and isoflavonoids in red propolis causes the inhibition of protozoa of the genus *Leishmania*, because these compounds alter the biosynthesis of sterols, decreasing the production of cholesterol and promoting the accumulation of ergosterol, changing the fluidity of the cell membrane, and causing toxicity to the parasite, a mechanism of change similar to that caused by the drug amphotericin B [34]. Chalcones can affect the cell viability of *Leishmania* species, acting on mitochondrial respiration, and inhibiting the activity of the mitochondrial dehydrogenase enzyme and fumarate reductase, specifically for these parasites [46]. 

To predict which of the identified substances may potentially contribute to the antileishmanial activity, we performed a docking analysis. Negative free-binding energies suggest that these interactions are favourable for the formation of ligand–receptor complexes. The root mean square deviation (RMSD) between the predicted docking conformation and the observed X-ray crystal structure of the ligand was 1.630 Å. Values below 2 Å indicate that the docking protocol is valid [25]. Through molecular docking, we discovered that among the compounds identified in the extract, liquiritigenin was the ligand that exhibited the most favourable parameters for complex formation with both CYP51 and TR. Consequently, this substance demonstrated the best antileishmanial action among the isoflavonoids tested.

Few studies report the biological activity of liquiritigenin; however, this compound has shown potential antioxidant and anti-inflammatory [47], anti-tumour [48,49], and in vitro activity against *Candida albicans*. It was successful in protecting mice against disseminated candidiasis in vivo, as it displayed immunomodulatory activity through the induction of Th1-type cytokine production from activated TCD4+ cells. Additionally, the intraperitoneal injection of 15 mg/kg/body weight of liquiritigenin in mice exhibited nephroprotective activity against cisplatin-induced acute kidney injury [50] and hepatoprotective and anti-liver fibrosis [51]. These findings are particularly encouraging since they demonstrate that a much higher concentration than the one used in our study was proven safe in experimental in vivo models. Furthermore, they emphasise the hepatic and nephroprotective properties of this compound, reinforcing its potential use for treating leishmaniasis, as the gold standard drugs currently employed are known to carry significant hepatic and nephrotoxic risks. Nevertheless, we propose that the observed biological effect in our study is related to the synergistic action of different compounds present in the sample. This is because other substances, such as calycosin and formononetin, which are present in larger quantities in the sample, also exhibited relevant leishmanicidal activity. 

We found no reported records of liquiritigenin activity against *Leishmania* in the literature. Therefore, the present study is the first to report the leishmanicidal potential of liquiritigenin.

## 5. Conclusions

Our findings indicate a new geographical origin of Brazilian red propolis, with a different main chemical marker than observed in red propolis from the northeast. We show here that calycosin is the major compound in this new variety of propolis. Furthermore, we demonstrate that Amazon red propolis has a high concentration of phenolic compounds and inhibited *L. amazonensis* promastigotes. All fractions exhibit leishmanicidal activity, with the most active fraction having a higher concentration of total phenolics and low toxicity to normal cells. We also report leishmanicidal activity from the flavonoids present in the sample, including liquiritigenin, calycosin, and formononetin. Future trials will be conducted to better understand the mechanism of action of these compounds on amastigotes, healthy cells, and experimental in vivo models. Additionally, it is necessary to identify its botanical origin.

## Figures and Tables

**Table 1:** Concentration of total phenolics and flavonoids of extracts and fractions obtained from Brazilian Amazon red propolis.

Sample	Total Phenolics (mg GAE/g)	Flavonoids (mg QE/g)
EHPV-1	309.27 ± 18.73 ^a^	34.38 ± 0.44 ^a^
EHPV-2	336.91 ± 8.77 ^b^	25.77 ± 0.94 ^b^
EHPV-3	192.03 ± 7.06 ^c^	16.70 ± 0.88 ^c^
EHPV-4	278.98 ± 10.00 ^d^	25.23 ± 0.57 ^b,d^
FrHX	77.33 ± 3.25 ^e^	nd
FrCL	328.44 ± 7.51 ^a,b^	25.46 ± 0.40 ^b,d^
FrEA	182.92 ± 2.77 ^c^	21.25 ± 0.08 ^d^

EHPV 1–4, Brazilian Amazon red propolis hydroethanolic extract; FrHX, n-hexane fraction of Brazilian Amazon red propolis; FrCL, chloroform fraction of Brazilian Amazon red propolis; FrEA, ethyl acetate fraction of Brazilian Amazon red propolis; nd, not determined. Results represent mean ± SD (*n* = 3); mg GAE/g-equivalent milligrams of gallic acid per gram of extract; mg QE/g-equivalent milligrams of quercetin per gram of extract. Different letters in the column indicate significant differences by Tukey test (*p* < 0.05).

**Table 2:** The leishmanicidal activity of Brazilian Amazon red propolis extracts and fractions against promastigote forms of *Leishmania amazonensis*.

Sample	IC_50_ (µg/mL)
EHPV-1	23.37 ± 1.4 ^a,e^
EHPV-2	31.38 ± 3.6 ^a,b^
EHPV-3	36.10 ± 3.7 ^b,c,d^
EHPV-4	34.31 ± 0.3 ^a,b,c,d^
FrHX	45.99 ± 5.2 ^d^
FrCL	16.11 ± 0.9 ^e^
FrEA	112.0 ± 7.8 ^f^
Amphotericin B	0.013 ± 0.1

EHPV 1–4, Brazilian Amazon red propolis hydroalcoholic extract; FrHX, hexane fraction of Brazilian Amazon red propolis; FrCL, chloroform fraction of Brazilian Amazon red propolis; FrEA, ethyl acetate fraction of Brazilian Amazon red propolis. Results represent mean ± SD (*n* = 3); IC_50_, concentration producing 50% inhibition of *L. amazonensis* promastigotes. Different letters in the column indicate significant differences by Tukey test (*p* < 0.05).

**Table 3:** Phenolic compounds identified in the Brazilian Amazon red propolis produced by *Apis mellifera*.

Peak	RT (Min)	[M + H]^+^ (*m/z*)	Product Ions MS/MS (*m/z*)							Tentative Identification	Relative Area (%)	
											EHPV-1	FrCL
1	23.1	301	284	269	255	241	213	137	123	7,8,3′-trihydroxy-4′-methoxyisoflavone	6.30	7.31
2	30.1	257	239	229	211	147	137			liquiritigenin ^a^	5.68	6.09
3	31.1	287	269	259	177	163	153	137	123	vesticarpan	1.95	1.84
4	36.4	317	299	289	207	179	163	135	107	violanone	1.13	1.31
5	37.1	287	255	241	193	177	153	147	123	3,8-dihydroxy-9-methoxy-pterocarpan	0.73	0.80
6	38.4	285	270	253	225	137	123			calycosin ^a^	21.87	23.78
7	42.5	287	269	259	177	161	153	139	137	3,4-dihydroxy-9-methoxy-pterocarpan	4.54	4.97
8	49.6	303	285	193	181	167	149	123	107	mucronulatol	1.49	1.73
9	50.6	301	269	241	191	167	147	123	107	3-hydroxy-8,9-dimethoxy-pterocarpan	0.65	0.72
10	58.9	273	163	151	137	123				vestitol	10.95	11.43
11	59.9	257	239	229	211	163	147	137		isoliquiritigenin ^a^	0.54	0.60
12	62.4	269	177	161	147	137	123			formononetin ^a^	12.72	14.42
13	63.7	271	243	229	177	161	147	137		medicarpin	3.30	1.83
14	77.7	285	270	253	181	163	137	123		biochanin A ^a^	0.31	0.44
15	83.9	523	399	387	373					retusapurpurin B	0.57	0.63

RT: Retention time; [M + H]+: positive mode ionization. EHPV-1: hydroalcoholic extract; FrCL: chloroform fraction. ^a^ Identification confirmed by comparison with standards.

**Table 4:** Free binding energies values obtained by molecular docking of the Brazilian Amazon red propolis compounds against *L. infantum* drug targets.

CYP51		TR	
Compound	ΔG_bind_ (kcal/mol)	Compound	ΔG_bind_ (kcal/mol)
liquiritigenin	−9.3	liquiritigenin	−8.9
calycosin	−9.3	7,8,3′-trihydroxy-4′-methoxyisoflavone	−8.2
7,8,3′-trihydroxy-4′-methoxyisoflavone	−9.1	biochanin A	−8.5
formononetin	−8.9	retusapurpurin B	−8.5
3,8-dihydroxy-9-methoxy-pterocarpan	−8.9	calycosin	−8.4
biochanin A	−8.8	vestitol	−8.4
medicarpin	−8.7	formononetin	−8.3
3,4-dihydroxy-9-methoxy-pterocarpan	−8.6	isoliquiritigenin	−8.1
vestitol	−8.4	3,4-dihydroxy-9-methoxy-pterocarpan	−8.1
violanone	−8.2	mucronulatol	−8.0
vesticarpan	−8.2	violanone	−7.7
isoliquiritigenin	−8.1	3-hydroxy-8,9-dimethoxy-pterocarpan	−7.2
mucronulatol	−7.9	medicarpin	−7.1
3-hydroxy-8,9-dimethoxy-pterocarpan	−7.5	3,4-dihydroxy-9-methoxy-pterocarpan	−7.1
retusapurpurin B	−6.2	vesticarpan	−7.0

## Data Availability

Not applicable.
